# Technical and Regulatory Shortcomings of the TaqMan Version 1 HIV Viral Load Assay

**DOI:** 10.1371/journal.pone.0043882

**Published:** 2012-08-24

**Authors:** Chanson J. Brumme, Luke C. Swenson, Brian Wynhoven, Benita Yip, Stuart Skinner, Viviane Dias Lima, Julio S. G. Montaner, P. Richard Harrigan

**Affiliations:** 1 BC Centre for Excellence in HIV/AIDS, St. Paul's Hospital, Vancouver, Canada; 2 Department of Medicine, University of Saskatchewan, Saskatoon, Canada; 3 Division of AIDS, University of British Columbia, Vancouver, Canada; McGill University AIDS Centre, Canada

## Abstract

**Background:**

The lower limit of detection of the original Roche Amplicor HIV plasma viral load (pVL) assay (50 copies/mL) has defined HIV treatment success. The Amplicor assay, however, has been replaced by the Roche TaqMan assay(s). Changes to the limits of detection and calibration have not been validated for clinical utility. Sudden increases in the number of patients with detectable pVL have been reported following the introduction of the TaqMan version 1 assay.

**Methods:**

Between October 2009 and April 2010 all routine pVL samples from British Columbia, Canada, with 40–250 copies/mL by TaqMan were re-tested by Amplicor (N = 1198). Subsequent short-term virological and resistance outcomes were followed in patients with unchanged therapy (N = 279; median 3.2 months follow-up).

**Results:**

TaqMan and Amplicor values correlated poorly at low pVL values. Low-level pVL by TaqMan was not associated with impending short-term virological failure; only 17% of patients with 40–250 copies/mL by TaqMan had detectable pVL by Amplicor at follow-up. During the follow-up period only 20% of patients had an increase in pVL by TaqMan (median [IQR]: 80 [36–283] copies/mL). In addition, in ∼2.4% of samples pVL was dramatically *underestimated* by TaqMan due to poor binding of the proprietary TaqMan primers.

**Conclusions:**

The replacement of Amplicor with the TaqMan assay has altered the previously accepted definition of HIV treatment failure without any evidence to support the clinical relevance of the new definition. Given the systematic differences in measurement in the low pVL range the British Columbia HIV treatment guidelines now use a threshold of >250 copies/mL by TaqMan to define treatment failure.

## Introduction

Highly Active Antiretroviral Therapy (HAART) has resulted in dramatic improvements in HIV outcomes [1], [2]. A direct measure of HAART success is an undetectable HIV plasma viral load (pVL). The Roche COBAS AmpliPrep/COBAS AMPLICOR HIV-1 MONITOR UltraSensitive Test, version 1.5 (“Amplicor v1.5”; Roche Molecular Diagnostics, Laval, Quebec, Canada) [3] was introduced for clinical use in British Columbia, Canada in 1999 and has been commonly used worldwide. International treatment guidelines have recommended that the goal of therapy is to achieve pVL below 50 copies/mL, the lower limit of detection of the Amplicor v1.5 assay [4], [5]. For over 10 years, an undetectable pVL by the Amplicor v1.5 assay was the *de facto* gold standard definition of therapy success in antiretroviral clinical trials and HIV treatment programs. However, the manufacture of the Amplicor v1.5 assay has been discontinued.

The Roche COBAS AmpliPrep/COBAS TaqMan v1 HIV-1 Test (“TaqMan v1”; Roche Molecular Diagnostics, Laval, Quebec, Canada) [6] has replaced Amplicor v1.5. While the assays are highly correlated over their dynamic range [7], a sudden increase in the number of patients with detectable pVL has been reported upon switching to TaqMan v1 [8]–[12]. These unexpected detectable pVL (typically 40–250 copies/mL) occurred among patients who had consistently suppressed pVL (<50 copies/mL) by Amplicor v1.5.

Sudden unexpected HIV detectability leads to additional pVL testing, and medical visits for re-evaluation of adherence, concomitant medications, drug resistance and comorbidities, resulting in significant additional costs and stress for patients, their support network, and health care providers [13].

We undertook the present study to compare the results of the previous Amplicor v1.5 and the replacement TaqMan v1 assays in cases of low-level viremia. First, we evaluate the concordance of the two assays when low-level pVL (40–250 copies/mL) are reported by TaqMan v1. Second, we evaluate short-term virological and antiretroviral resistance outcomes in patients with low-level detectable TaqMan v1 pVL receiving stable HAART. Third, we show that the TaqMan v1 assay may also systematically *underestimate* pVL in a minority of cases due to imperfect binding of the primers and/or probes.

**Figure 1:**
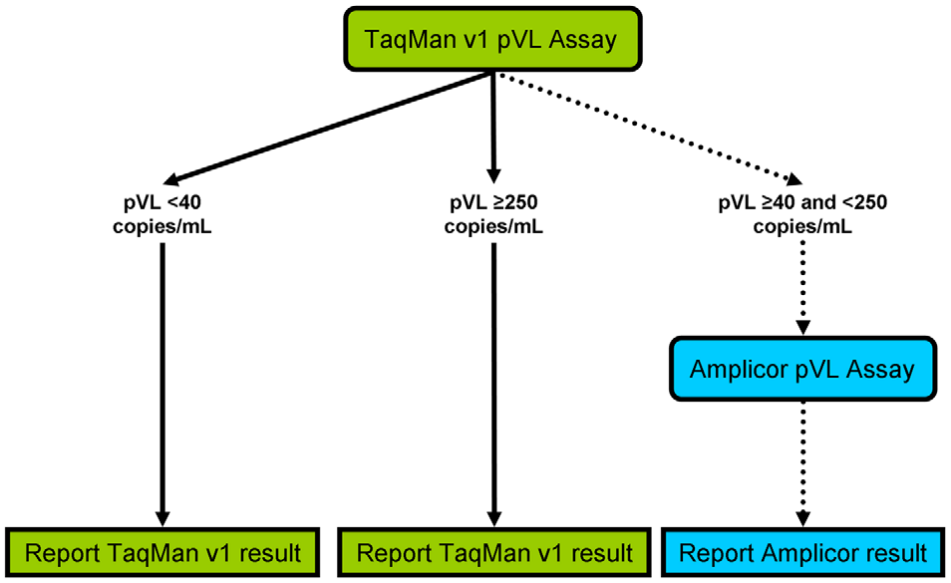
Plasma viral load testing and reporting protocol in British Columbia between October 2009 and April 2010. All plasma samples were initially tested with the Roche COBAS AmpliPrep/COBAS TaqMan v1 HIV-1 Test (“TaqMan v1”) assay. TaqMan v1 pVL results <40 or ≥250 copies/mL were reported to physicians. Samples with TaqMan v1 pVL ≥40 and <250 copies/mL were re-tested by the Roche COBAS AmpliPrep/COBAS AMPLICOR HIV-1 MONITOR UltraSensitive Test, version 1.5 (“Amplicor v1.5”). The Amplicor v1.5 test results were reported to physicians.

## Methods

The TaqMan v1 assay was introduced in British Columbia (BC), Canada in February 2008. After observing a high number of patients with newly detectable viral loads by the new assay and poor concordance of pVL results <100 copies/mL from both assays [8] a new testing protocol was implemented. In brief, between October 2009 and April 2010 samples having a TaqMan v1 pVL result ≥40 and <250 HIV RNA copies/mL were re-tested using the Roche Ultrasensitive Amplicor v1.5 assay. In these patients, only the results of the re-testing with Amplicor v1.5 were reported to the prescribing physicians, as this was the standard of care in BC prior to the introduction of the TaqMan v1 assay (Figure 1). HIV viral load measurements were undertaken as per the specific test kit instructions [3], [6]. The TaqMan v1 assay was performed either on fresh (for locally-collected samples) or previously frozen (for samples requiring shipment) plasma aliquots. Prior to re-testing by Amplicor v1.5, samples were stored at −20°C until TaqMan v1 pVL results were available. The concordance between TaqMan v1 and Amplicor v1.5 values was assessed in samples with low-level pVL (40–250 copies/mL). The analysis presented is therefore limited to a one-way comparison between low-but-detectable TaqMan v1 pVL results and their corresponding Amplicor v1.5 results. Due to the limited availability of Amplicor v1.5 pVL test kits following its discontinuation, a two-way comparison could not be performed.

Subsequent short-term virological and resistance outcomes were followed in patients who started HAART at least 6 months prior and who did not change therapy regimens during follow-up (N = 279 patients). HIV RNA detectability by TaqMan v1 and Amplicor v1.5 were compared at the latest follow-up timepoint up until April 2010. Antiretroviral drug resistance genotyping was performed for all follow-up samples with viral loads >250 copies/mL by TaqMan v1, as described elsewhere [14].

Re-testing samples with Amplicor v1.5 revealed a subset of samples (N = 29) with systematically *underestimated* viral loads by TaqMan v1 compared to Amplicor v1.5. For these patients, archived samples were re-tested with the Roche TaqMan version 2 (“TaqMan v2”) [15] and/or the Abbott *m*2000 RealTi*m*e HIV-1 assay (Abbott Molecular Diagnostics, Wiesbaden, Germany). Where sufficient plasma was available, HIV gag was also sequenced to assess potential TaqMan v1 primer incompatibility.

Ethical approval for this study was granted by the Providence Health Care/University of British Columbia Research Ethics Board (H10-01778). The requirement for individual consent was waived by the Research Ethics Board as the study involved no more than minimal risk to subjects.

## Results

Between October 2009 and April 2010 a total of 1198 samples (from 950 patients) with pVL 40–250 copies/mL by TaqMan v1 were re-tested with the Amplicor v1.5 assay. This comprised approximately 10% of all tests performed in British Columbia during this period. In BC ∼97% of antiretroviral-treated patients are infected with subtype-B HIV-1 [16].

**Figure 2:**
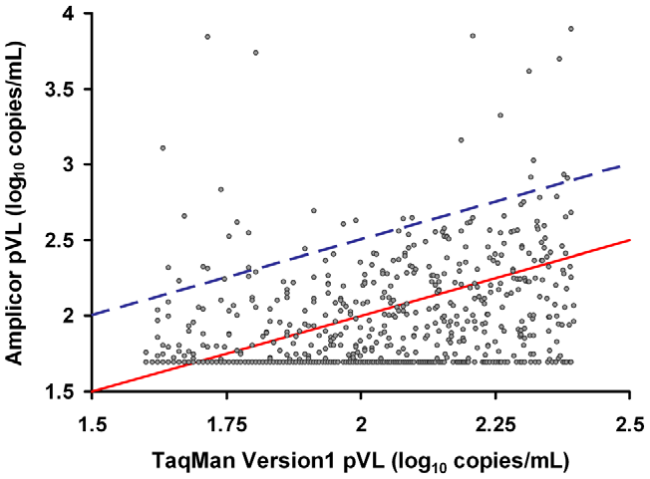
Poor concordance between TaqMan v1 and Amplicor v1.5 at low plasma viral load levels (40 –**250 HIV RNA copies/mL by TaqMan v1).** Between October 2009 and April 2010 plasma samples from British Columbia with low-level viremia (40–250 copies/mL) by the Roche TaqMan v1 assay (N = 1198) were systematically re-tested by the Amplicor v1.5 assay. Poor concordance was observed between TaqMan v1 and Amplicor v1.5 results, with 82% of values falling below the line of identity (red solid line). Points above the blue dashed line are samples in which TaqMan v1 *underestimated* pVL by ≥0.5 log_10_ copies/mL relative to the Amplicor v1.5 assay.

The concordance between the two viral load assays was poor at low viral load strata (Figure 2). The median (interquartile range) pVL was 79 (54–124) and <50 (<50–74) HIV RNA copies/mL by the TaqMan v1 and Amplicor v1.5 assays, respectively. This difference was statistically significant (Wilcoxon signed-rank test; p<0.0001). Visual inspection of the Bland-Altman plot (Figure S1) suggests a bias towards higher pVL results obtained by TaqMan v1 in this range. Of all the samples with detectable pVL by TaqMan v1, 66% were undetectable by Amplicor v1.5 (789/1198 samples). If only the 965 samples with pVL ≥50 copies/mL by TaqMan v1 were considered, only 385 (40%) had detectable pVL by Amplicor v1.5. Overall, the majority of samples tested (82%, 984/1198) fall below the line of identity in Figure 2, suggesting that TaqMan v1 reports pVL that are consistently higher than results obtained by Amplicor v1.5 in this range.

**Figure 3:**
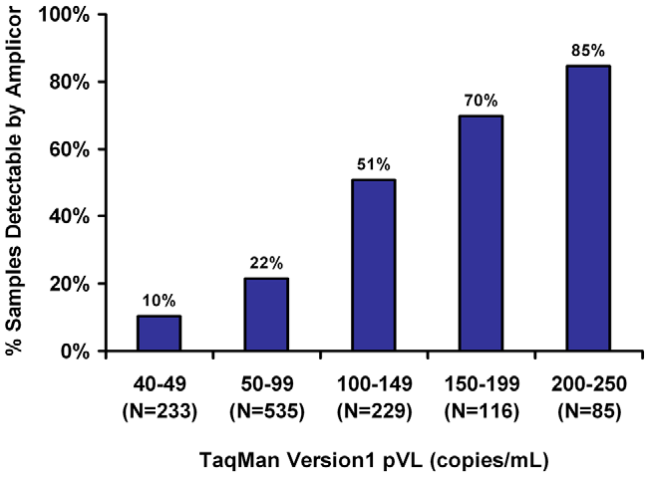
The proportion of samples detectable by Amplicor v1.5 increases as a function of the TaqMan v1 value. Of 1198 samples with pVL results 40–250 copies/mL by TaqMan v1, only 34% were detectable when re-tested by Amplicor v1.5 (>50 copies/mL). When TaqMan v1 results were grouped into 50 copies/mL strata we observed a stepwise increase in detectability by Amplicor v1.5 with increasing viral load by TaqMan v1.

Despite the low concordance between the two assays, there was a stepwise increase in detectability by Amplicor v1.5 with increasing pVL by TaqMan v1 (Figure 3). Samples with TaqMan v1 values 50–99 copies/mL were detectable (≥50 copies/mL) by Amplicor v1.5 in 22% of cases (116/535 samples), while samples with TaqMan v1 pVL 200–250 copies/mL were detectable by Amplicor v1.5 in 85% of cases (72/85 samples).

### Detectability by TaqMan v1 does not predict short-term virologic failure or antiretroviral resistance

**Figure 4:**
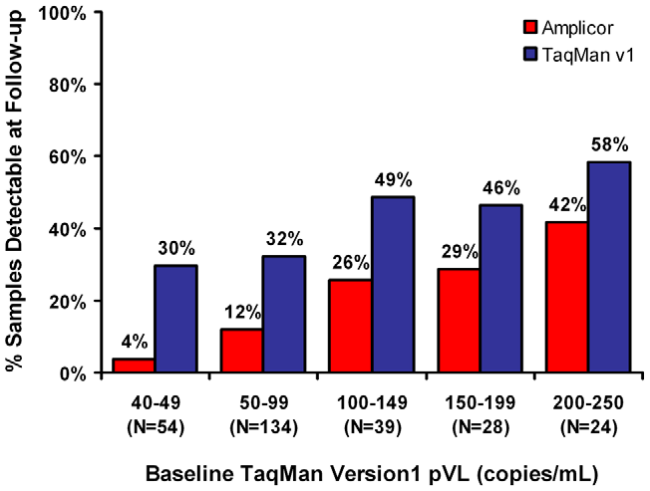
Low-level viremia by TaqMan v1 does not predict short-term virological failure. A subset of patients (N = 279) with low-level viremia (40–250 copies/mL) by TaqMan v1 were followed longitudinally for a median of 3.2 months (IQR: 2.0–4.2 months). Patients initiated HAART at least 6 months prior, and treatment regimens remained unchanged over the course of follow-up. Samples from patients' latest follow-up visit were re-tested with the TaqMan v1 and Amplicor v1.5 assays. Overall 17% of patients had a detectable viral load by Amplicor v1.5 at their latest follow-up visit, while 38% were detectable by TaqMan v1. When patients were grouped according to their baseline TaqMan v1 pVL into 50 copies/mL strata we observed a stepwise increase in the proportion of patients with detectable pVL at their latest follow-up visit by both Amplicor v1.5 (red bars) and TaqMan v1 (blue bars). Consistent with previous results, more patients had detectable pVL by TaqMan v1 than by Amplicor v1.5 at follow-up in all strata.

To determine whether low-level TaqMan v1 pVL is a predictor of emergent virologic failure, we followed a subset of patients treated with stable HAART. A total of 279 of 950 (29%) eligible patients with low-level viremia by TaqMan v1 and remaining on a constant regimen were followed for a median of 3.2 months (IQR: 2.0–4.2 months). A median of one (IQR: 1–2) follow-up pVL test per patient was performed. Note that during this period, the Amplicor v1.5 pVL results were used to guide therapy decisions. Baseline patient characteristics are shown in Table 1. At baseline, 31% of these patients had a detectable pVL when re-tested by Amplicor v1.5. Overall, 59 (21%) of patients had a least one pVL ‘blip’ ≥50 copies/mL by Amplicor v1.5 during follow-up, compared to 126 (45%) by TaqMan v1. At the latest follow-up timepoint, 17% of patients had detectable pVL by Amplicor v1.5, whereas 38% of patients had detectable pVL by TaqMan v1. Furthermore, as baseline TaqMan v1 viral loads increased, the proportion of patients with detectable pVL at follow-up by both assays also increased (Figure 4). Overall, 20% of patients had an increase in pVL by TaqMan v1 (median pVL increase [IQR]: 80 [36–283] HIV RNA copies/mL) during the follow-up period. This suggests that the higher pVL results obtained by TaqMan v1 relative to Amplicor v1.5 are systematic and not merely the result of increased viral ‘blips’. Overall, the median viral load of all patients in this cohort decreased over the follow-up period from 75 (IQR <54–121) copies/mL at baseline to <40 (IQR <40–74) copies/mL at the last follow-up timepoint.

**Table 1:** Patient characteristics of 279 HAART-treated patients with low-level viremia followed longitudinally.

Variable	N (%) or Median (IQR)
Male gender	226 (81%)
Age at HAART initiation (years)	40 (33−47)
History of injection drug use	126 (45%)
Baseline CD4 cell count (cells/mm^3^)	390 (230−530)
Baseline plasma viral load by TaqMan v1(HIV RNA copies/mL)	75 (53–121)
Baseline plasma viral load by Amplicor v1.5 (HIV RNA copies/mL)	<50 (<50–68)
Previous therapy experience (months)	54.2 (21.1−94.6)
Follow-up from baseline to last visit (months)	3.2 (2.0−4.2)

IQR: Interquartile Range.

Follow-up samples collected prior to September 2010 with viral load >250 copies/mL by TaqMan v1 were also tested for antiretroviral resistance using standard genotypic methods (N = 66 successfully tested of a total of 69 patients). A median of one genotype per patient (IQR 1–2) was obtained over a median of 4.4 (IQR: 0.9–6.9) months. New resistance mutations in protease/reverse-transcriptase (PR-RT) were observed in eight patients (12%) compared to pre-therapy sequences, suggesting that, in general, low-level pVL by TaqMan v1 may not be indicative of emerging resistant variants. It is notable that only three patients with newly-detected resistance mutations had undetectable viral loads by Amplicor v1.5 at their baseline visit.

### Mismatches in the TaqMan v1 primer binding regions in HIV *gag* result in systematic *underestimation* of viral load in a subset of patients

**Figure 5:**
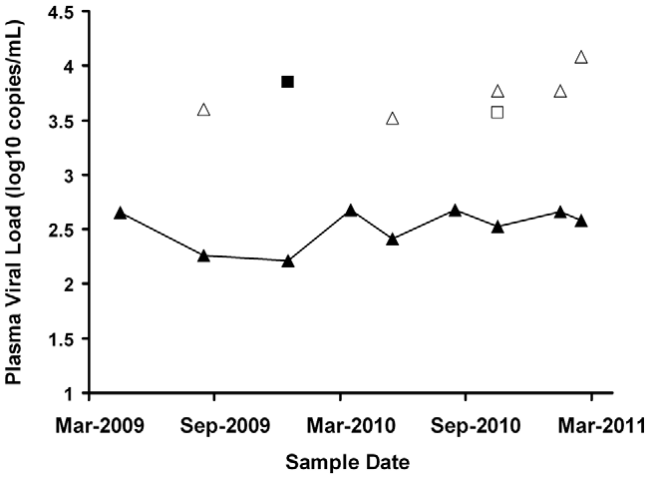
Systematic underestimation of plasma viral load by TaqMan v1 in a minority of patients. A minority (2.4%) of samples had viral loads *underestimated* by >0.5 log_10_ copies/mL by TaqMan v1 after re-testing with Amplicor v1.5. Depicted here is the viral load history of one representative patient showing systematic underestimation of viral load by TaqMan v1 when compared to results obtained by re-testing samples with the Amplicor v1.5, TaqMan v2 and/or Abbott assays. TaqMan v1 viral load results are shown as solid triangles (▴) joined by a solid line. Overlaid are the corresponding results from the Amplicor v1.5 (solid squares ▪), TaqMan version 2 (unshaded triangles Δ) and Abbott (unshaded squares γ) assays where available. For this patient TaqMan v1 systematically under-reported pVL by an average of 1.3 log_10_ copies/mL over a period of 18 months.

In addition to the frequent pVL overestimation reported by TaqMan v1, a small subset of samples tested (∼2.4%, 29/1198) had pVL levels by TaqMan v1 >0.5 log_10_ *lower* than by Amplicor v1.5 (Figure 2). For these patients, archived plasma samples with low TaqMan v1 pVL (range: <40–458 copies/mL) were re-tested using Roche COBAS AmpliPrep/COBAS TaqMan HIV-1 Test, v2.0 (“TaqMan v2”; Roche Molecular Diagnostics, Laval, Quebec, Canada) (N = 31) and/or the Abbott *m*2000 RealTi*m*e HIV-1 assay (“Abbott”; Abbott Molecular Canada, Toronto, Ontario, Canada) (N = 15) if sample volume permitted. Of 18 samples with undetectable pVL by TaqMan v1, five (28%) were detectable by TaqMan v2 (range 81–382 small subset of samples tested copies/mL). In 13 samples with low but detectable pVL by TaqMan v1, TaqMan v2 results were a median of 1.1 log_10_ copies/mL *higher* (IQR: 0.87–1.34 log_10_ copies/mL), consistent with a systematic *underestimation* of the pVL levels by TaqMan v1 in a specific subset of patients (Figure 5). This observation was not restricted to samples from BC. One sample collected in Saskatchewan with a reported “undetectable” pVL by TaqMan v1 had a confirmed a pVL level >200,000 copies/mL by other tests (data not shown). All patients with underestimated pVL were infected with HIV-1 subtype B.

We hypothesized that the underestimation of pVL results by the TaqMan v1 assay was due to inefficient binding of the HIV *gag* assay primers. To explore this issue further, we sequenced HIV-1 *gag* in samples exhibiting low TaqMan v1, but high Amplicor v1.5 pVL. As the sequences of the TaqMan v1 assay primers are unpublished, *gag* sequences were sent to Roche (Laval, Quebec) for interpretation. Of the nine *gag* sequences shared with Roche, seven (78%) apparently had mutations potentially incompatible with the TaqMan v1 primers: two (22%) and five (56%) showed incompatibilities with the “upstream” and “downstream” primers, respectively. Since the TaqMan v1 primer and probe sequences remain proprietary, we cannot report the specific mutations involved.

New resistance mutations were detected in one patient sample out of seven with longitudinal PR-RT sequences available. In this sample, viral load was underestimated by 2.5 log_10_ copies/mL as compared to TaqMan v2.

## Discussion

The introduction of a new HIV viral load assay (“TaqMan v1”) in February 2008 resulted in an unexpected increase in the prevalence of detectable HIV loads in British Columbia, Canada [8]. When samples with low but detectable pVL (40–250 copies/mL by TaqMan v1) were re-tested using the Amplicor v1.5 assay, we found a poor concordance between the values, with close to two-thirds of samples giving undetectable pVL by Amplicor v1.5. It is highly unlikely that this sudden change in pVL detectability is due to differences in assay performance in non-B HIV subtypes, as the HIV-1 epidemic in British Columbia consists overwhelmingly of subtype B infections [16]. Our data clearly demonstrate that a pVL <50 by Amplicor v1.5 is not equivalent to <50 small subset of samples tested copies/mL by TaqMan v1. Therefore, an unintended consequence of replacing the Amplicor v1.5 assay with the TaqMan v1 assay was that it altered the nearly universally accepted definition of virological failure. Approximately 6% of all pVL tests performed in BC between October 2009 to April 2010 had false-positive results using the previous criteria.

The clinical consequences of low-level viremia were unclear even before the TaqMan v1 assay was instituted. Some studies have indicated that intermittent low-level viremia is associated with a higher risk of virological failure or drug resistance [17], [18] and have found associations between the risk of failure and the magnitude of viral ‘blips’ [19], [20]. However, other studies have failed to find such links [21]. To evaluate the management of patients on HAART monitored with the TaqMan v1 assay, we prospectively followed a subset of these patients for a period of approximately 3 months while on stable therapy. Physicians were informed of only Amplicor v1.5 pVL values, so patients who had undetectable pVL by Amplicor v1.5 but detectable pVL by TaqMan v1 did not receive any additional adherence counseling or pVL monitoring. A full 83% of these patients had undetectable pVL by the Amplicor v1.5 assay at follow-up, and had little evidence of drug resistance evolution. The median TaqMan v1 pVL in this group decreased over the follow-up period with 62% having a pVL <40 copies/mL at their latest follow-up visit. Unfortunately, the limited availability of Roche Amplicor v1.5 assay kits following their discontinuation restricted parallel testing by TaqMan v1 and Amplicor V1.5 to a relatively narrow range of pVL (40–250 copies/ml) and a short follow-up time.

New resistance mutations were observed in only 12% of tested patients with pVL >250 copies/mL by TaqMan v1 over the course of follow-up. However, it should be noted that uncertainty and stochastic variation in testing samples with low viremia could result in resistance mutations being missed or detected by chance in some cases. Nevertheless, these results suggest that low but detectable pVL <250 copies/mL by TaqMan v1 do not correlate with detectability by Amplicor v1.5, and are not indicative of impending short-term virological failure or drug resistance. Rather, we estimate that on average a pVL value of 150 copies/mL by TaqMan v1 is approximately equivalent to a pVL value of 50 copies/mL by Amplicor v1.5. However, it is important to note that the reverse may not be true – an Amplicor v1.5 pVL value of 50 copies/mL may not be equivalent to a TaqMan v1 value of 150 copies/mL as this scenario was not evaluated. While this is not sufficient to entirely repudiate the validity of the TaqMan v1 assay, there exists no positive evidence to support that TaqMan v1 pVL levels of 40–250 copies/mL are clinically relevant. Given the change in assays, the definition of “virological failure” should be re-validated against clinically meaningful parameters; the British Columbia HIV treatment guidelines have revised the definition of treatment failure as two consecutive pVL measurements >250 copies/mL by TaqMan [22]. Similarly, the treatment guidelines from the US Department of Heath and Human Services now define virological failure as a pVL rebound to >200 copies/mL following previous suppression [23]. In contrast, other studies have suggested that detectable pVL below the limit of quantification may be predictive of viral rebound, and therefore the definition of virological failure be adjusted downwards [24]. The definition of virological failure in the treatment guidelines of the European AIDS Clinical Society, and the International AIDS Society-USA remains unchanged.

This study also confirmed an unrelated but potentially more serious shortcoming of the TaqMan v1 assay. The TaqMan v1 primers were unable to efficiently amplify their targets in a subset of ∼2.4% of samples tested leading to the systematic underestimation of viral loads by up to 2.5 log_10_ copies/mL as confirmed by re-testing by Amplicor v1.5, TaqMan v2, and/or the Abbott RealTi*m*e assays (see also [9], [15], [25]). When HIV-1 *gag* sequences from these samples were sent to Roche for analysis, 78% were identified as having mutations incompatible with the TaqMan v1 primers. Of concern, as the TaqMan v1 primer and probe sequences remain unpublished, there is no mechanism available to predict or to retrospectively identify which patients belong to this subset. However, if the locations and sequences of the primers were made available, HIV-1 *gag* could be genotyped for primer incompatibilities in patients suspected of having systematically underestimated pVL. The consequences of incorrectly diagnosing an undetectable pVL in a pregnant woman or the HIV-positive partner in a serodiscordant couple, for example, could be disastrous. Unfortunately, the study design did not permit the accurate quantification of the proportion of “false-negative” results (i.e. an “undetectable” pVL by TaqMan v1, but pVL >50 copies/mL by Amplicor v1.5). Due to the limited availability of Amplicor v1.5 kits in Canada following its discontinuation only a one-way analysis could be performed.

The discrepancies between Amplicor v1.5 and TaqMan v1 pVL at low-level pVL may have arisen due to the methods used to validate the TaqMan v1 assay. Validation was performed by testing TaqMan v1 results against the Amplicor v1.5 assay over a very wide range of pVL. No special attention was placed on pVL in the clinically-relevant range (<1000 copies/mL), where the test results are directly used to guide therapeutic decisions [4], [6]. Validating TaqMan v1 with pVL nearing 10^6^ copies/mL may have obfuscated clinically meaningful differences between the two assays. Therefore, we recommend that in the future new or “upgraded” pVL assays be validated with a particular focus on the clinically-relevant range before commercial approval.

An updated version of the TaqMan v1 assay (version 2.0) is now available. While the assay upgrade appears to have corrected the systematic underestimation of pVL occasionally seen in TaqMan v1 [26], version 2.0 may still show higher pVL quantitation than Amplicor v1.5 in the lower range of detection; nearly 12% of samples with an undetectable pVL by Amplicor v1.5 still have a pVL result >50 copies/mL by TaqMan v2 [12]. Other higher-sensitivity HIV pVL assays with decreased lower limits of quantification, such as the Abbott m2000 RealTime assay, are in routine clinical use elsewhere. Although these assays have not been evaluated here, given further investigation they may represent alternatives to the Roche TaqMan system. However, regardless of the pVL assay used, the uncertainty surrounding the clinical relevance of low-level viremia requires that a set of technical and regulatory standards to evaluate the clinical utility of new diagnostic assays be rigorously defined.

## Supporting Information

Figure S1 **Bland-Altman plot of results from parallel testing of viral load samples by TaqMan v1 and Amplicor v1.5.** Visual inspection of the plot suggests a bias towards higher pVL results obtained by TaqMan v1 at low pVL (40–250 copies/mL). When attempting to interpret these results it is important to note that 1) Amplicor v1.5 pVL values below the limit of quantification (<50 copies/mL) were coded as 49 copies/mL, and 2) the plotted results are restricted to samples with *TaqMan v1* results 40–250 copies/mL.
(TIF)
