# Supplementary figures and images for: Technical and Regulatory Shortcomings of the TaqMan Version 1 HIV Viral Load Assay
Source: PLoS One. 2012 Aug 24;7(8):e43882. doi: 10.1371/journal.pone.0043882 (PMC3427308; doi:10.1371/journal.pone.0043882)

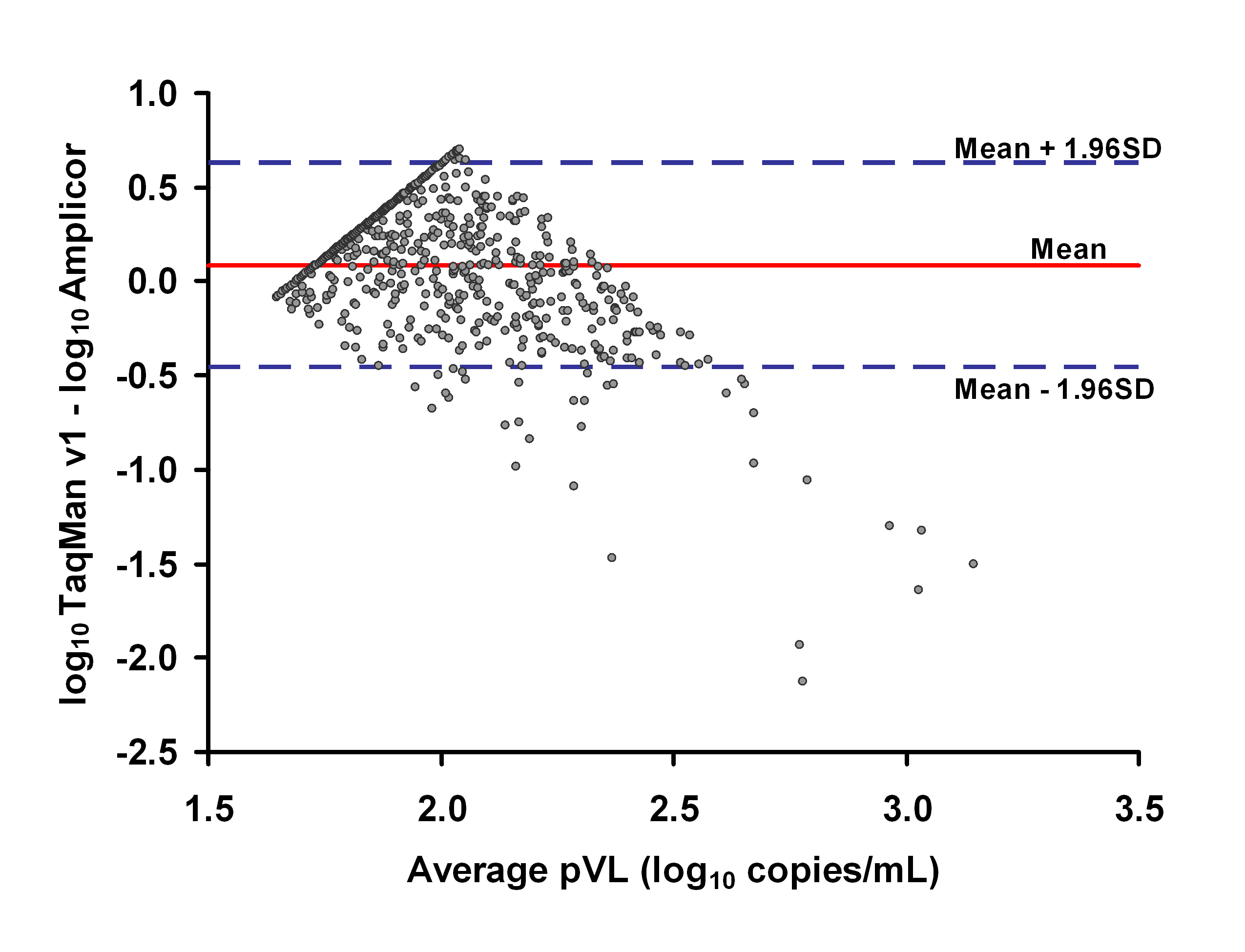

Supplement: Figure S1 — Bland-Altman plot of results from parallel testing of viral load samples by TaqMan v1 and Amplicor v1.5. Visual inspection of the plot suggests a bias towards higher pVL results obtained by TaqMan v1 at low pVL (40–250 copies/mL). When attempting to interpret these results it is important to note that 1) Amplicor v1.5 pVL values below the limit of quantification (<50 copies/mL) were coded as 49 copies/mL, and 2) the plotted results are restricted to samples with TaqMan v1 results 40–250 copies/mL. (TIF) [file pone.0043882.s001.tif]
